# Philosophical impact of psychosurgery: a narrative of the history of psychosurgery

**DOI:** 10.1192/j.eurpsy.2023.2166

**Published:** 2023-07-19

**Authors:** J. Wellington, K. Miller, M. Wallace, A. Smith, T. Spelman, K. Walters, A. Yang, F. Davis

**Affiliations:** Cardiff University School of Medicine, Cardiff, UK, United Kingdom

## Abstract

**Introduction:**

To fully comprehend and appreciate the impact of psychosurgery on treatment-resistant depression it is pertinent to review its initial development and subsequent history. By reviewing previous studies of psychosurgery, we can build a narrative of what was, what currently is and what might be. Assessing the complex philosophical dilemma of the mind and the impact this has on individuals’ concept of psychosurgery has helped to bridge the gap between Neurosurgery and Psychiatry.

**Objectives:**

We aimed to examine this question, starting at the very beginnings of our concept of mind, working through to modern-day thinking, how we approach both neurosurgery and psychiatry and help to bridge the two.

**Methods:**

A narrative review of the current literature concerning neurosurgery for mental disorders and said applications to modern psychiatry was conducted. Emphasis on philosophical thought processing in conjunction with the neurosurgical intervention was noted.

**Results:**

Psychosurgery has its roots in the early philosophy of mind, concerned with distinguishing whether the mind is a physical entity or immaterial. Psychosurgery is reliant on a physical concept of the mind, or at the very least that the mind supervenes the physical brain. History has shown us examples of this, with the archetype of this being the story of Phineas Gage. Since its onset psychosurgery has moved in and out of vogue. After being met with early scepticism it later went on to be performed thousands of times to help cure schizophrenia. In the 1800s, Gottlieb Burkhardt pioneered initial surgical interventions on the brain with intended psychiatric outcomes, moving on to work from Egas Moniz and the development of leucotomies and famously lobotomies, to modern medical techniques of Deep Brain Stimulation.

**Conclusions:**

Psychosurgery has faced much opposition throughout history due to the uniquely invasive nature of not just affecting us physically but also mentally and the implications that this has for us as humans and our understanding of ourselves. As both medical and cultural views of mental health have changed over time, so has our understanding of psychosurgery and its potential applications. It is possible that early attempts to implement psychosurgery, before the advent of modern medicine, did more harm to psychosurgery’s reputation than good. However, without those early forays, we may never have progressed to the modern techniques we now utilise.

**Disclosure of Interest:**

None Declared

